# Simplified “on‐couch” daily quality assurance procedure for CT simulators

**DOI:** 10.1120/jacmp.v10i3.2844

**Published:** 2009-06-17

**Authors:** Ruijie Rachel Liu, Karl Prado, Michael Gillin

**Affiliations:** ^1^ Department of Radiation Physics The University of Texas M. D. Anderson Cancer Center Houston Texas USA

**Keywords:** computed tomography simulator, imaging quality assurance, laser quality assurance, fat couch top, quality assurance phantom

## Abstract

For most computed tomography (CT) simulators, radiation therapists must first remove the fat couch top in order to perform daily CT quality assurance (QA), and then use separate tools to perform localization‐laser QA. This process wastes time and effort, and creates the opportunity for accidents to occur. In this study, we tested a simple, yet comprehensive, daily QA program and phantom designed for CT simulators used in radiation oncology that would enable us to use only one tool to perform both laser and imaging QA on a fat couch. To construct a modified QA phantom, we attached three adjustable legs and fastened two metric scales (one vertically and one horizontally) to a commercial CT QA phantom. The adjustable legs helped to position and level the phantom conveniently in the needed position. The two metric scales were used for localization‐laser QA, while the phantom body was used for CT imaging QA. We evaluated five different CT scanners from two manufacturers with their designated couches to evaluate this phantom system. Since the couch is scanned along with the phantom, we evaluated the couch's effect on image quality. We found that the presence of the couch top changed the uniformity of water's CT number slightly, but did not change the visual image resolution. The couch top also produced different, yet reproducible, effects on image quality. The effects were greatest in the section of the phantom closest to the couch top. For a commercial carbon fiber couch top, the variation was within 3 Hounsfield Units (HU). The effect was couch‐ and scanner‐specific, and could be incorporated into the QA acceptability criteria for each CT scanner. By using the proposed QA program and phantom, we have been able to implement more thorough QA while decreasing the amount of effort and time the simulation therapists spend performing laser and imaging QA.

PACS number(s): 87.55.Gh, 87.55.Qr, 87.56.Fc, 87.57.Q‐

## I. INTRODUCTION

Computed tomography (CT) scanners have been designed primarily for diagnostic imaging, in which the patient lies on a curved couch. Daily imaging quality assurance (QA) for a CT scanner uses a phantom that is attached to the end of this curved couch. Since a fat couch is needed for radiotherapy CT simulators to simulate a treatment couch, most CT manufacturers include a fat top in addition to the curved couch for applications in radiation oncology. For daily QA, therapists must remove the fat couch top, mount the CT QA phantom to the curved couch, and then perform imaging QA. After imaging QA, therapists must remove the phantom, replace the fat top, and use a separate tool to perform localization laser QA. Because the couch is heavy, switching couches not only wastes time and effort, but may also cause accidents or damage to the facility.

To avoid removing the fat couch to perform CT QA and to simplify the daily QA procedure while performing more thorough QA, we designed a simple yet comprehensive daily QA program and phantom for CT simulators that encompasses both laser and imaging QA. While conventional imaging QA uses “phantom in‐air” CT imaging, our new procedure scans the couch and the phantom concurrently. Therefore, we needed to evaluate the couch's effect on image quality and incorporate it into the QA criteria. The purpose of this study was to demonstrate the feasibility of this CT QA method.

## II. MATERIALS AND METHODS

**Figure 1:**
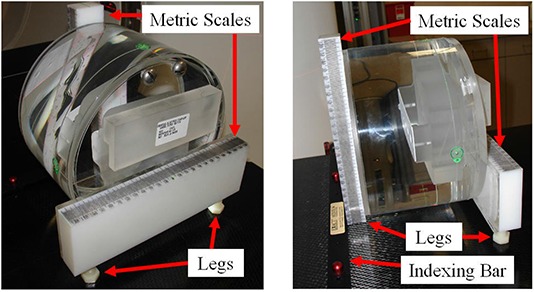
Modified on‐couch daily quality assurance (QA) phantom.

To construct a new daily QA phantom (Fig. 1), we modified a General Electric (GE) CT QA phantom (GE Medical Systems: LightSpeed 5.X Technical Reference Manual, Chapter 12 “Quality Assurance”. 2003. Milwaukee, WI) by attaching three adjustable legs and fastening two metric scales, one vertically and one horizontally, to the phantom ends. The adjustable legs stabilized the phantom in the needed position. We use the two metric scales for laser QA, and the phantom body for CT imaging QA. The phantom includes three imaging sections, each having a 21 cm diameter: high‐contrast resolution, low‐contrast resolution, and uniformity (water). To measure CT caliper accuracy, we placed three high‐attenuating nonlead bead markers with 2.3 mm diameters (CT‐SPOTS pellets; Beekley, Bristol, CT) on the left, right, and top of the phantom in the high‐contrast resolution section of the phantom.

For conventional in‐air phantom measurements, we attached to the couch end unmodified QA phantoms used with CT scanners from both GE and Philips Medical Systems (Bothell, WA), following the manufacturers’ recommended procedures for daily QA setup. The scanners and their couches are described below. It should be noted that the GE scanners’ QA phantoms were the same model as our modified on‐couch phantom, whereas the Philips scanner's QA phantom (Philips Medical Systems: Mx8000 Operation Manual, Appendix B “Image Performance Quality Assurance”. 2002. Highland Heights, OH) consisted of a head section of 20 cm diameter and a body section of 30 cm diameter. The head section contains subsections of imaging features and water, and therefore was used for evaluation. The modified on‐couch phantom and procedure was compared with conventional laser and imaging QA using five CT scanners and phantoms and their respective couch tops: GE LightSpeed (LS) RT (4‐channel, 80‐cm bore), GE LS RT 16 (16‐channel, 80‐cm bore), GE LS 16 (16‐channel, 70‐cm bore), GE 8‐channel PET/CT (16‐channel, 70‐cm bore), Philips MX 8000 IDT 16 (16‐channel, 70‐cm bore). MedTec fat tops (CIVCO, Orange City, Iowa) were used on all couches. We also tested the MedTec foot extension of the GE LS RT 16.

**Figure 2:**
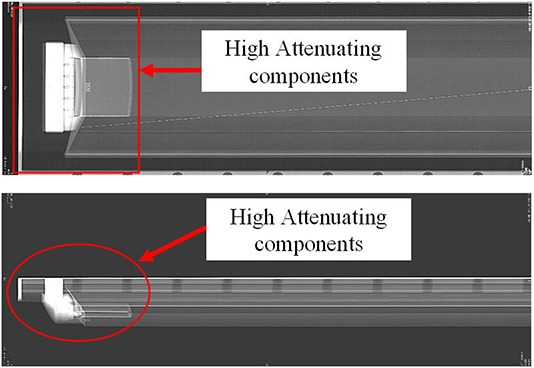
Scout views of a GE LightSpeed RT CT couch with MedTec fat top: (a) 0° view; and (b) 90° view.

The couches were scanned first with pilot views (GE names it “scout view”). Figure 2 shows an example of the pilot views of the couch at gantry 0° and 90°, acquired with the GE LS RT scanner using a 120 kVp X‐ray beam. The pilot views revealed an attenuation pattern of the couch with a fat top, which was similar for all scanners and their couches. High‐attenuating components were found at the couch end. To avoid using the high‐attenuating section, we positioned the on‐couch QA phantom close to the middle of the couch. To ensure consistently reproduced images, we used “an indexing bar” to help place the phantom at a designated couch position. Phantom legs, left‐right position, and couch height were adjusted so that the phantom was positioned at isocenter as conventional QA setup.

First, we scanned the phantoms with clinical‐equivalent techniques as follows: 120 kVp, 250 mAs, head mode, 25‐cm display FOV, 2.5 mm (GE) or 3 mm (Philips) image thickness. Then we scanned the water section of the phantom with close‐to‐maximum technique and greatest image thickness in order to reduce the quantum noise level and to see the couch effect on image uniformity. For GE scanners, we used 1760 mAs and 10 mm image thickness. For Philips MX8000, we used 750 mAs and 6 mm image thickness. We configured maximum detector channels and beam width in the multichannel CT scanners. Two QA procedures were performed for each scanner: the conventional QA setup and the on‐couch QA setup. For the Philips Mx8000, we scanned the modified on‐couch GE phantom and compared its images to those of the head section of the Philips’ QA phantom in air. The high‐contrast resolution, low‐contrast resolution, and uniformity of all the GE QA images acquired with clinical techniques were evaluated and compared. Images acquired with maximum technique were used for uniformity evaluation. The uniformity was measured in the water section by the mean HU value using regions‐of‐interest (ROI) with a size of about 168 mm^2^ (automatically generated ROI size by the GE CT scanner software), placed at the center, then the 12, 3, 6, and 9 o'clock positions. For the periphery ROIs, the ROIs were placed about one ROI diameter in from the edge of the phantom. Differences in uniformity between phantoms in‐air and on‐couch were used to evaluate the couch effect.

## III. RESULTS

**Figure 3:**
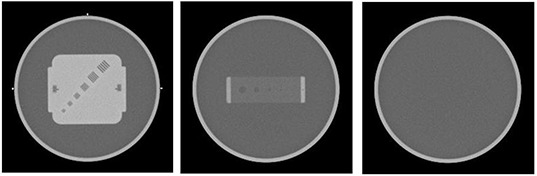
Imaging quality assurance (QA) sections used for evaluation: (a) high‐contrast and scale (markers attached on the phantom side); (b) low‐contrast; and (c) uniformity (water).

**Figure 4:**
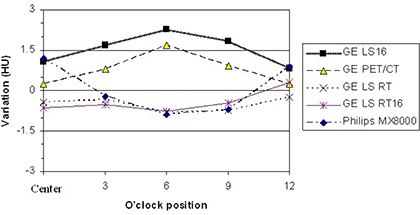
Difference in CT number of water between in‐air and on‐couch phantoms. Horizontal axis: zero indicates center position; the other “o'clock” positions indicate corresponding periphery positions.

We evaluated high‐contrast resolution (Fig. 3(a)) and low‐contrast resolution (Fig. 3(b)) for GE scanners, and found that they were, within the sensitivity of the phantom, equivalent to phantom in‐air QA as performed with unmodified phantoms attached to the couch end. Similarly, the presence of the couch had no effect on CT‐scale accuracy, determined by measuring the distance between the lateral bead markers (Fig. 3(a)). The couch, however, did produce changes in the measured CT number and uniformity of water (Fig. 3(c)). In Table 1, the highlighted cell indicates the location where the variation between the on‐couch QA and the in‐air QA was greater than 3 HU, which occurred at 6 o'clock position for the foot extension only. The differences between in‐air and on‐couch settings were plotted in Fig. 4 for couches with the regular MedTec fat tops.

**Table 1:** Changes in CT number in water for phantoms in‐air and on‐couch top.

*CT Scanners*	*Phantom Position*	*Measured CT # at various positions (HU*)					
		*Center*		*O'clock positions at edge: Mean*			
		*Std. Dev.*	*Mean*	*12*	*3*	*6*	*9*
GE LS RT	In‐air	1.28	1.89	1.62	1.74	1.86	2.01
	On‐couch	1.34	1.45	1.37	1.41	1.06	1.28
GE LS RT 16	In‐air	1.38	−0.33	0.26	0.28	−0.06	0.08
	On‐couch	1.58	−0.39	−0.03	−0.24	−0.83	−0.37
	Foot extension	1.73	1.77	0.63	1.31	9.84	0.76
GE PET/CT	In‐air	1.26	−0.39	−0.38	−0.55	−0.50	−0.45
	On‐couch	1.30	−0.15	−0.12	0.25	1.17	0.48
GE LS 16	In‐air	1.29	−0.21	0.05	0.34	0.38	0.10
	On‐couch	1.35	0.86	0.87	2.01	2.64	1.94
Philips MX 8000	In‐air	2.3	−1.4	−1.5	−2.0	−1.6	−1.7
	On‐couch	2.7	−0.2	−0.6	−2.2	−2.5	−2.4

The maximum difference in CT number was generally greatest in the section of the phantom closest to the couch (the 6 o'clock position) and the minimum difference occurred at the 12 o'clock position. The effects were scanner‐ and couch‐specific. The CT number of water varied by no more than 3 HU for the regular MedTec fat tops. The couch top's presence increased the CT number for GE LS scanners with a regular bore (70‐cm diameter), but only slightly decreased the CT numbers for the GE LS RT systems with a large bore (80 cm or larger in diameter). The maximum CT number difference of on‐couch versus in‐air is 2.3 HU for the regular fat tops. For maximum techniques, the pixel standard deviation (SD) of water is around 1.3 HU for GE scanners, and 2.3 HU for the Philips scanner. For clinical techniques, the pixel SD of water is around 7 HU for GE scanners, and 6 HU for Philips scanners. Because of its increased attenuation, the foot extension, which was placed on the fat couch top, caused larger variation in CT number in the GE LS RT 16 scanner. For the Philips Mx8000, the maximum difference of 1.2 HU occurred at center; the CT numbers at the center and 12 o'clock position increased, while others decreased.

## IV. DISCUSSION

Due to the beam hardening effect, it is expected that the presence of additional objects in the scan FOV would change the CT number and uniformity of the phantom images. Our modified on‐couch phantom caused little CT number variation because the couch and its attached fat top had low attenuation, except at the couch end. As was shown in the GE LS RT 16‐foot extension, large couch attenuation had the largest effect on CT number at the 6 o'clock position relative to other positions.

The presence of the couch in the CT field of view causes small – yet measurable and reproducible – changes in CT number. These observed changes in CT number may be significant. The standard uncertainties of the mean^(1)^ CT numbers in the ROIs that were used to characterize CT number were of the order of 0.1 HU. (When quantum noise dominates, the SD of the $\text{mean}=\text{pixel}\;\text{SD}\;/\;\sqrt{N}$, where SD=Standard Deviation and N=the number of pixels in the ROI). In most cases, the changes in mean CT number caused by the couch exceeded this level, lending some credence to our assumption that couch effects are significant and need to be included in QA criteria.

This method allows us to do more comprehensive QA according to the standards set in AAPM Task Group Report 66^(2)^ with less time and effort. We give two examples of daily QA procedures performed on GE LS 16 and RT in Table 2. Note that the uniformity criteria were set differently for both scanners.

**Table 2:** Examples of CT daily QA procedure and criteria: GE LS 16 and RT.

*GE LS*	*X Laser Accuracy* (±1mm)	*Z Laser Accuracy* (±1mm)	*High‐contrast Resolution & X‐spatial Integrity*		*Low‐contrast Resolution*	$1^{\text{st}}$ *Water Image CT# & Uniformity*		*Other Water Images CT# & Artifact*	
*RT*	±100	±100	Bars (≥4)	BB distance (217±2mm)	Holes (≥4)	Center ±3HU	12 o'clock ±3HU	Center ±3HU	Artifact present?
*GE LS*	*X Laser Accuracy* (±1mm)	*Z Laser Accuracy* (±1mm)	*High‐contrast Resolution & X‐spatial Integrity*		*Low‐contrast Resolution*	$1^{\text{st}}$ *Water Image CT# & Uniformity*		*Other Water Images CT# & Artifact*	
*16*	±100	±100	Bars (≥4)	BB distance (217±2mm)	Holes (≥4)	Center ±3HU	12 o'clock ±3HU	Center ±3HU	Artifact present?

The on‐couch QA phantom and procedure we investigated here is just an example. It is not limited to any given manufacturers’ phantom. Other widely used commercial phantoms, such as Catphan 500 (The Phantom Laboratory, Inc., Salem, NY)^(^ ^3^ ^–^ ^4^ ^)^ or the American College of Radiology (ACR) CT accreditation phantom,^(5)^ can be used as well to build the on‐couch QA phantom. The method of modifying the phantom and establishing QA criteria should be the same.

For those CT models that can mount the QA phantoms at the end of the fat couch tops, such as the Philips AcQsim and the Briliance 64, our method does not save as much effort as is the case with scanners that need to have the fat couch tops removed to perform QA. Even for those scanners, however, this method still has the advantage of ease of positioning and the ability to check lasers simultaneously. The couch end mounting method does not allow using the phantom to check the wall and ceiling lasers because the couch cannot be moved back that far. Our on‐couch phantom does not have this limitation.

## V. CONCLUSIONS

We have used this program for the GE scanners for more than a year. The feedback from the therapists has been very positive. They report that not only does not having to mount and dismount the fat couch top save time, but it also simplifies phantom positioning compared with the usual couch‐end mounting. For the on‐couch procedure, once the phantom legs were adjusted initially, no further adjustment was needed. We were able to consistently reproduce the phantom's position with the same couch height and longitudinal couch position.

Because of mishandling, we have had to adjust the phantom legs for two GE scanners twice since we initiated this program. Placing a warning sign that described correct handling of the phantoms helped prevent this. Our modified on‐couch phantom was more trouble‐free than the couch‐end phantom; it was sometimes dropped or occasionally the attaching handle detached during mounting. Use of the on‐couch phantom prevents all these issues. Usually, however, the modified on‐couch phantom cannot be mounted to the couch end. Therefore, we suggest that institutions keep a regular manufacturer's QA phantom for service and annual calibration tests.
